# Kangaroo Mother Care in Term and Late Preterm Neonates: A Systematic Review

**DOI:** 10.7759/cureus.60958

**Published:** 2024-05-23

**Authors:** Ravi Gajula, Greeshma Reddy Kankanala, Ragini Mutukulla, Rakesh Kotha

**Affiliations:** 1 Pediatrics, Government Medical College, Siddipet, Siddipet, IND; 2 Neonatology, Osmania Medical College, Hyderabad, IND

**Keywords:** term neonates, kangaroo mother care, hypothermia prevention, pain scores, serum bilirubin levels, late preterm neonates

## Abstract

This systematic review aims to investigate the efficacy of kangaroo mother care (KMC) in term and late-preterm babies. Based on the Preferred Reporting Items for Systematic Reviews and Meta-Analyses (PRISMA) recommendations, seven studies were analyzed, which covered a wide range of outcomes, from the post-vaccination serum bilirubin level and pain during the vaccination to the prevention of hypothermia and long-term neurodevelopmental outcomes. Results point out that KMC might come with some advantages such as the reduction of neonatal bilirubin levels, a painless and quicker vaccination process, and better prevention of hypothermia. Moreover, initial and lengthy KMC also plays a possible role in the better long-term brain development of low-birth-weight neonates. Furthermore, the limitation of smaller numbers of studies and variability in results remains to be solved. The next step is working to build stronger evidence and creating proper conditions for the implementation of KMC in future healthcare.

## Introduction and background

Kangaroo mother care (KMC) has been shown to be effective in neonatal care, especially for preterm infants, during skin-to-skin holding with the mother or caregiver. First designed in a resource-vulnerable situation to combat the shortcomings of preterm birth, KMC has now gained momentum in the global health community for the benefits it brings to neonatal and pediatric outcomes. Traditionally, it has been considered to be done mainly for preterm infants, but there is a rising interest in exploring its adaptation to term and late preterm neonates as well. Neonatal care, particularly for premature babies, is a very complex yet elegant task that demands a multidisciplinary approach. Preterm birth, which refers to births before the 37th week of gestation, is one of the major global problems relevant today [1]. 

The KMC model shows a new way of caring for babies by focusing not only on the medical aspects but also on the emotional needs of the infant and mom. The techniques involve putting the infant in an upright position on a mother's chest with skin-to-skin contact between the mother's clothing or a special garment. The mother's body heat helps maintain the infant's temperature, which is enough to reduce any risk of hypothermia, and her close physical proximity to the infant facilitates cuddling, breastfeeding, and physiological stability [2].

Despite the fact that KMC has been widely studied and proven effective in preterm babies, its application in term and late preterm children is still not clearly understood. Term newborns, who are born between 37 and 42 weeks of gestation, and late preterm newborns, who are born between 34 and 36 weeks of gestation, constitute the majority of newborns all over the world [3]. These infants can also benefit from the physiological and psychological benefits of KMC but the effectiveness of KMC in this population is not yet clear. On the other hand, awareness of the possible advantages and disadvantages of using KMC in cases of term and late-preterm neonates is the basis for improving the practices of neonatal care. Through a review of the existing evidence and the areas of ignorance, healthcare professionals can adjust interventions to fit the particular requirements of this population. Not only this, but the disclosure of the mechanisms responsible for the advantages of KMC in term and late preterm neonates can lead to the development of interventions and policies dedicated to the improvement of neonatal outcomes all over the world.

## Review

Methods

**Figure 1:**
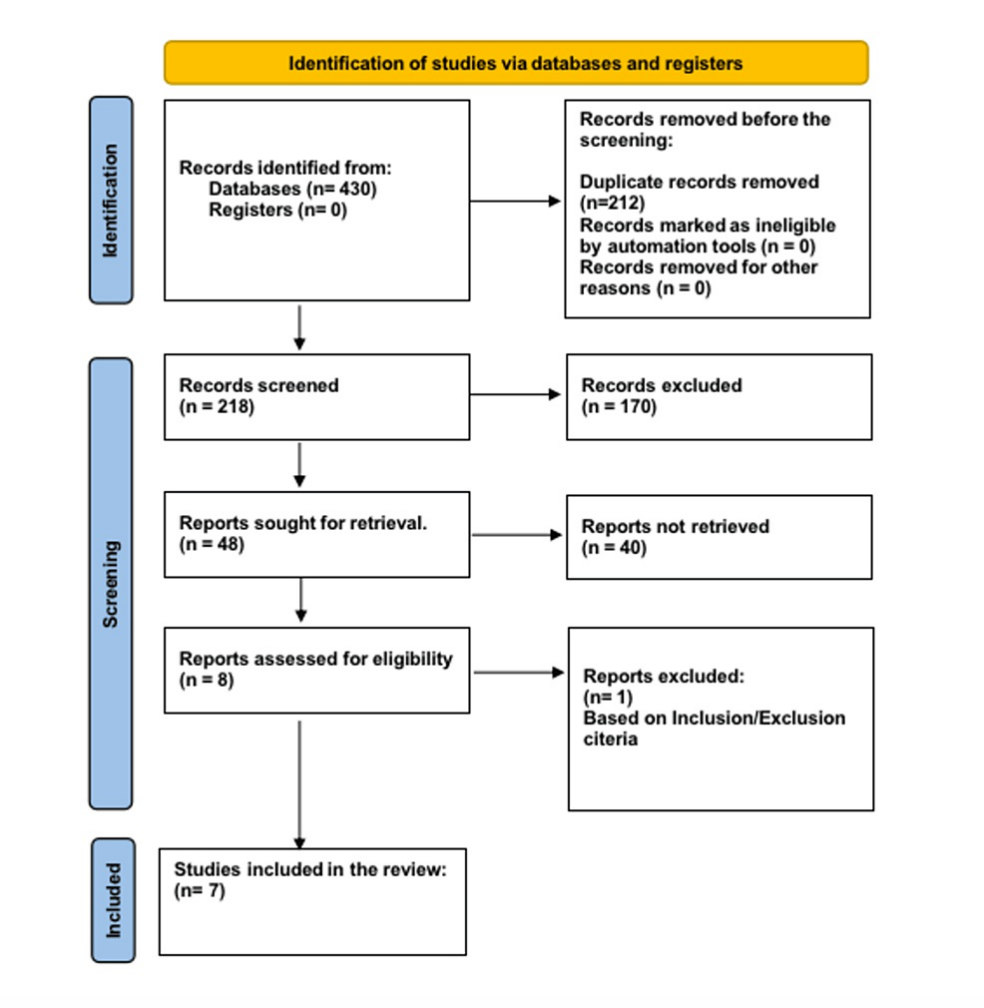
PRISMA diagram for identification of studies PRISMA: Preferred Reporting Items for Systematic Reviews and Meta-Analyses

This systematic review adhered to a systemized, high-quality methodology, following the Preferred Reporting Items for Systematic Reviews and Meta-Analyses (PRISMA) guidelines (Figure 1). It was registered in the International Prospective Register of Systematic Reviews (PROSPERO) (registration number: 548048).

A thorough review was conducted of different electronic databases including PubMed, Embase, and Scopus to find relevant studies published during 2017-2023. Keys like "Kangaroo Mother Care," "term neonates," and "late preterm neonates," and their synonyms were used so the search engines could maximize the search's relevance (Table 1). The inclusion criteria comprised randomized controlled trials, clinical trials, cohort studies, and observational studies in which the impact of the KMC on full-term and late-preterm neonates was the main focus. Studies devoted to a number of outcomes such as serum bilirubin level, pain scores, hypothermia prevention, and later neurodevelopmental issues were included (Table 2). The screening procedure was conducted by the two different reviewers, and when there were any disputes, those were settled by reaching a consensus [4]. Data extraction involved categorization under the following headings: study characteristics, participant demographics, interventions, outcomes, and significant findings. Quality assessment was made using the right instruments in accordance with the study types that were taken into consideration in order to avoid any possible bias.

**Table 1:** Search Strategy

Search	Strategy
#1	"Kangaroo Mother Care" AND "term neonates"
#2	"Kangaroo Mother Care" AND "late preterm neonates"
#3	"Kangaroo Mother Care" AND "neonatal outcomes"
#4	"Kangaroo Mother Care" AND "serum bilirubin levels"
#5	"Kangaroo Mother Care" AND "Vaccination Pain Scores"
#6	"Kangaroo Mother Care" AND "Hypothermia Prevention"
#7	"Kangaroo Mother Care" AND "Neurodevelopmental outcomes."

**Table 2:** Selection criteria

Inclusion Criteria	Exclusion criteria
Randomized controlled trials, clinical trials, cohort studies, and observational studies	Case reports, case series, and case studies.
Studies examining the efficacy of kangaroo mother care (KMC) in term and late preterm neonates	Studies focusing solely on preterm neonates without term neonates
Studies published between 2017 and 2023	Studies with insufficient data or unclear methodology
Studies reporting outcomes such as serum bilirubin levels, pain scores, hypothermia prevention, and long-term neurodevelopmental outcomes	Studies with conflicts of interest or funding bias
Studies with clear intervention protocols and outcome measures	

Quality Assessment of the Studies

AMSTAR (A Measurement Tool to Assess Systematic Reviews) is a valuable tool for assessing the methodological quality of systematic reviews and meta-analyses within the medical research field [5-7]. The authors of the methodological AMSTAR have made it their aim to provide the field with a tool for criticizing the quality of the systematic reviews that is both rigorous and reliable. It includes a set of 11 indicators representing the various criteria that should be assessed in each case, for example, experimental design, study selection, data harvesting, and statistical treatment. With every product assessed, "Yes," "No," or "Can't Answer" is the score awarded on the basis of prepared criteria in advance. These criteria incorporate features like the predefined research design, duplication in the process of selecting literature, analysis of the data, comprehensive literature search, identification of publication bias, and disclosure of conflicts of interest.

The identification of the appropriate and highest quality of the studies done by appropriate quality assessment tools.. Furthermore, other than the Cochrane risk-of-bias tool, which facilitates judging the key domains where applicable, an appraisal instrument for the assessment of these domains was developed that encompasses sequence generation-randomized and allocation concealment, blinding of personnel and participants, incomplete outcome data, selective reporting, and other biases. We used the Newcastle-Ottawa Scale (NOS) to assess cohort studies or observational studies. It measures the quality and representativeness of groups or cohorts, compares similar groups, and, lastly, evaluates outcomes like exposure. This undertaking employed specific criteria to assess both the data quality and research methods of each study. The NOS rates the selected articles in three categories: good, bad, and worst [8]. All articles selected were identified as being of good quality according to the NOs. Thus, factors producing the bias and the confounding could be later analyzed. At this stage, the studies with non-conformance or doubts were identified and discussed among the group to identify discrepancies. An unbiased decision was made when selecting the studies for the systematic review. As per our assessment, all studies were of high quality on a particular scale

Results

The details of the included studies are given in Table 3.

**Table 3:** Summary of the selected studies

Study	Type of Study	Year	Country	Objective	Comparative Group	Conclusion/Outcome
Lori Kenari et al. [9]	Systematic Review	2020	Iran	Assessing the effect of kangaroo mother care on serum bilirubin levels in neonates	Field massage	Kangaroo mother care was associated with a significant reduction in serum bilirubin levels among neonates under phototherapy
Fallah et al. [10]	Interventional Study	2017	Iran	Evaluating the impact of kangaroo mother care, breastfeeding, and swaddling on vaccination pain	Breastfeeding	Kangaroo mother care significantly reduced Bacillus Calmette-Guerin vaccination pain scores in term neonates
Ramani et al. [11]	Randomized Controlled Trial	2018	Zambia	Investigating kangaroo mother care for preventing neonatal hypothermia in term neonates	Standard care	Kangaroo mother care effectively prevented hypothermia in term neonates
Garg et al. [12]	Systematic Review	2020	India	Assessing the role of kangaroo mother care in managing neonatal hyperbilirubinemia	No intervention	Kangaroo mother care demonstrated benefits in managing hyperbilirubinemia in both term and late preterm neonates
Bisanalli et al. [13]	Cohort Study	2023	India	Examining the long-term neurodevelopmental outcomes of early and prolonged kangaroo mother care	Standard care	Early and prolonged kangaroo mother care was associated with improved long-term neurodevelopmental outcomes in low birth weight neonates
Karnati et al. [14]	Review Article	2020	Global	Discussing the changing trends and challenges in managing late preterm infants	None	Late preterm infants present ongoing challenges in neonatal care, necessitating continuous adaptation and improvement
Arelly et al. [15]	Observational Study	2019	Pakistan	Assessing the impact of kangaroo mother care on weight gain and breastfeeding in late preterm neonates	Standard care	Kangaroo mother care promoted better weight gain and increased breastfeeding rates among late preterm neonates

Ariff et al.'s trial was clustered, randomized, and controlled in order to evaluate the associated risk of neonatal mortality for late preterm and preterm infants within the rural areas of Pakistan [3]. Their study proposed that KMC should be factored into the initial stages of neonate intervention. Besides being feasible and cost-effective, this will reduce neonatal mortality rates in low-resource settings [16]. This study attempts to narrow knowledge gaps on KMC scalability by evaluating KMC programs that improve neonatal mortality in these settings and helping the global community apply the findings.

Lori Kenari et al.'s study was a comparison of KMC and field massage on serum bilirubin of the term neonates with hyperbilirubinemia while they are on phototherapy in a neonatal intensive care unit (NICU) [9]. The objective of their study was to determine the efficacy of KMC and light massage as adjuvant therapies in conjunction with phototherapy to treat hyperbilirubinemia in term newborns. Similarly, weight gain and early feeding are effective adjuvant therapies for reducing newborn jaundice in preterm and late-preterm neonates [17,18].

Fallah et al. organized a clinical trial that compared the extent of pain induced by the Bacille Calmette-Guerin (BCG) vaccination after instituting KMC, breastfeeding, and swaddling in healthy term neonates [10]. The study is meant to understand if KMC is effective in minimizing pain during vaccination, not only when compared to other care methods but also during breastfeeding. This study, through the analysis of accompanying pain scales, sought to determine which technique is more suitable to effectively help infants from that age group with pain mitigation during BCG vaccination as well as improve the overall vaccination experience for both children and caregivers.

Ramani et al. conducted a randomized trial with a controlled balance to find out whether there is a KMC authenticity that prevents neonatal hypothermia in term neonates [11]. The trial evaluated if KMC during the first six months of life makes neonates with a weight of 2.5 kg less likely to develop hypothermia. Through determining the significance of KMC related to term neonates' body temperature stability, this study sought to help clinicians in particular devise better policies and guidelines for ways of using KMC to prevent the incidence of hypothermia among term neonates, which might help in improving the rates of healthy survivors.

While the systematic review by Garg et al. focused on KMC for neonatal hyperbilirubinemia in both term and late preterm infants [12], it still remains interesting to explore ways of managing this condition in those who are not offered this care. We aim to critically review existing evidence that proves noninvasive KMC is a novel care intervention for properly managing the hyperbilirubinemia of newborns. Their review focused on the search and examination of similar findings from multiple studies and then gave an overview regarding the effectiveness of KMC in maintaining bilirubin levels in serum (normal) and preventing complications related to hyperbilirubinemia, which can be useful for practitioners and future researchers in the field [12].

While skin-to-skin contact strengthens the bond between mother and child, involving the family and men will further strengthen it [19,20]. Bisanalli et al.'s study showed that the beneficial effect of early and prolonged effective KMC on neurodevelopmental outcomes in late preterm and low-birthweight babies at later times is significant [13]. Their review explored the neurodevelopmental outcomes of KMC over an extended period, which brought up a lot of important information concerning the long-term benefits of KMC for late preterm neonates and clinical practice, and guidelines on the implementation of KMC in neonatal wards in care settings was one of them [13].

Karnati et al., in their review article, evaluated the changes in the trend of late preterm infant care and the enduring obstacles to this treatment [14]. Knowledge of the specific physiology of late preterm infants is important to be able to adopt tailored approaches to their treatment [21]. The present review tries to draw conclusions related to the current state of the issue as well as identify the ongoing challenges. It aims to inform healthcare professionals about the peculiar needs of late preterm infants and advocate for the allocation of additional resources, improvement of the target interventions, and development of guidelines to improve outcomes for this vulnerable population.

Discussion

The included studies jointly expand the ever-increasing corpus of knowledge about the effect of KMC and its subsequent outcomes on a wide range of newborn issues. KMC, a low-cost and easy-to-implement intervention that involves the transmission of warmth between the mother and the baby through skin-to-skin contact, has triggered deliberate discussions about the efficiency of the intervention for improving neonatal health and a consequent drop in neonatal mortality and morbidity rates, which is more noticeable in preterms and low birthweight infants.

These studies demonstrate a wide variety of objectives, methods, and populations and cover a wide range of research in the field of KMC. While some studies concentrate on specific situations like neonatal hypothermia or hyperbilirubinemia, others look at the general effect of KMC on psychomotor development and vaccination pain score [22]. The reviewed articles also include various study designs such as randomized control trials, patient care surveys, cohort studies, and systems reviews. Such a diverse nature of studies indicates that they reveal the positive effects of KMC not only in one health indicator of the newborn but in many. Moreover, the investigations, including those of Ariff et al. [3] and Ramani et al. [11], prove that KMC has the ability to help stabilize the temperature of newborns and lower mortality rates in the case of late preterm and low-birth-weight infants [16,11]. Much like Garg et al. point out, KMC is concerned with neonatal hyperbilirubinemia management [12]. Additionally, Bisanalli et al. argue that KMC provides long-term benefits in relation to neurodevelopment, which is particularly important in late preterm and low-birth-weight babies [13].

There are findings that show how KMC has contributed to enhancing the health of infants in the community and in hospitals [16]. Pradhan et al. evaluated the applicability of KMC as a complementary treatment in the hospital setting [23], while Ariff et al. [3] and Bisanalli et al. [13] examined the role of KMC in rural populations in the community setting, with results showing favorable outcomes for newborns in the longer term.

Researchers may try to identify strategies for addressing the diverse barriers and bottlenecks of effective KMC implementation in the varied sectors. Karnati et al. skip this topic and instead go straight to the trends and problems underlying the care of late preterm infants [14]. Developing multidisciplinary care strategies for KMC babies plays a major role in understanding, as defined by Lawn et al. [4]. This would be the way to deal with the vastly complicated needs of the population.

Keeping in mind the various studies reviewed, it can be said that KMC is a comprehensive and cost-effective strategy that can be replicated for different populations and settings, and is an effective way to improve universal neonatal healthcare. While that is the case, some more studies are needed to explore the suitable procedures for implementation and the long-term effects. Taneja et al. mentioned there was no neurodevelopmental effect on late-preterm and term-small gestational-age babies by KMC at one year of age. However, they too suggested that some more studies and long-term follow-up should be done to explore the long-term effects [24]. This will help nurses, physicians, policymakers, and researchers to develop strategies for practicing and policymaking, and research in neonatal care can be gradually improved.

Comparison to Other Studies

There are frequent reports of KMC succeeding in diverse neonatal outcomes, such as reducing infant mortality, preventing hypothermia, increasing breastfeeding, and creating life-long bonding between the mother and the baby. Studies have clearly revealed that KMC is more efficacious in lowering rates of mortality and morbidity among preterm and low-birth-weight babies relative to conventional nursing [3,4]. These findings also emphasize that KMC positively affects neonatal health.

The studies that are incorporated in this review evaluate the relative efficacy of KMC to different interventions or to empirical treatment. For instance, Fallah et al. compare outcomes emanating from KMC, breastfeeding, and swaddling in healthy babies [10], while Lori Kenari et al. look at the incidence of KMC compared to field massage impact on serum bilirubin level of term neonates with hyperbilirubinemia [9]. Likewise, as Ariff et al. portray, one of the ways of delivering KMC packages in a rural Pakistani community where resources are limited is through the comprehensive involvement of the community itself [3]. In the same way, Bisanalli et al. elucidate the long-term neurodevelopmental consequences of KMC in neonates of low birth weight [13]. They suggest evaluating the broader implications of KMC treatment, stretching beyond the immediate health outcomes.

The studies featured in this review provide useful information regarding the effectiveness and feasibility of KMC. While these studies form a sound basis for support, they are substantiated by a body of literature that is equally credible, showing that the use of KMC is both cost-effective and evidence-based in improving neonatal health outcomes [25]. This kind of approach allows researchers and policymakers alike to come up with a holistic understanding of the advantages and avenues of KMC and eventually devise a way to improve its delivery and impact on both global and grassroots levels.

Limitations of a Systemic Review

Systematic reviews that are designed to summarize a body of evidence and guide clinical practice also have their drawbacks. Knowing these limits is a prerequisite for understanding the implications of the systematic review results. The major limitation of this particular review is the inadequate literature; nonetheless, we included a wide range of studies. Below, we mentioned the general limitations of a systematic review [26].

Publication bias: There is a research discrepancy in terms of the most likely publishing of the studies with statistically significant results and those without significant findings, which may lead to a greater exaggeration of the effectiveness of treatment methods. Such bias can lead to weakening the validity of a systematic review.

Search bias: Although exhaustive search schemes may be in place, a systematic review could still be incomplete primarily due to poor database coverage, language setbacks, or a lack of systematic indexing. As a result, the review might not do the literature justice by taking a holistic view of the available evidence. So, the conclusions and recommendations drawn from the review might be biased.

Selection bias: Systematic reviews may unintentionally form a selection bias if the researcher has defined criteria that automatically exclude certain types of studies. The selective exclusion of studies that have smaller sample sizes or use non-randomized designs, among other instances, can reduce the generalizability of the review's conclusions and undermine its ability to provide a comprehensive synthesis of the evidence.

Heterogeneity: Variability from the original nature of the subjects (mobility), the type of intervention (e.g., drug or surgery), the intended outcome (improvement or preventative), or the methodology (experimental or observational) used in the studies may be sources of heterogeneity when evaluating these studies in a review. Meta-analysis is not only a way to identify or assess heterogeneity, but it may also be the main reason why the findings of reviews or systematic reviews differ from each other, and, as a result, the robustness of final results may lose efficacy.

Quality of included studies: The research quality of one systematic review may be extremely different from the next. A few studies use poorly designed methods, incorrect ways of conducting a study, or have different biases, and it can complicate our analysis and prevent generalization. The criteria for the appraisal of the quality of the results, as well as the mechanisms for determining the sources of biases, directly affect the outcome and lead to the evidence that will be used for the decision-making process.

Risk of confounding: Some systematic reviews may ignore some of the confounders leading to risk from different diseases, co-existing conditions, or different drug-drug combinations across the studies. Many factors in comparative studies are related to the outcome of the review. Sometimes we miss evaluating some factors and therefore may get misleading results.

Publication time lag: In a systematic review, sometimes the duration needed to collect data, analyze the data, and publish the results to give recent evidence, which requires recent research, may be very long. Because of this, the time of publication may be delayed. Hence, the knowledge in the discipline may not be up-to-date after the publication, as envisioned. For these reasons, such a long period may lead to misconceptions and doubts about the reliability of the results.

## Conclusions

This systematic review documented the effectiveness of KMC in terms of the benefits to term and late preterm newborn infants in different clinical settings. The evidence from the different studies has shown that KMC plays an important role in maintaining neonatal health benefits, which include prevention of hypothermia, reduction of high levels of bilirubin, alleviation of the pain resulting from vaccination, and development of the brain in the long term. Nevertheless, it is worthwhile to note that the review has inherent shortcomings such as publication bias, search bias, and the heterogeneity of the studies that were included in it. Despite the limitations mentioned, the data emphasizes the significance of the KMC as normal care used for term and late preterm neonates to improve their condition and development.

Moving forward, research should be done to fill the gaps in the literature, which can lead to stronger and more effective evidence for the implementation of KMC in neonatal care settings. Eventually, considering KMC as a core part of total neonatal service care systems has the power to drastically change the level of service and the health outcomes for newborns and their families. Randomized controlled trials in term and late preterm neonates in other areas like oxygen dependency duration in meconium aspiration syndrome and the role of KMC in developmental disorders are needed.
